# Impact of Modified Triple Salt Monolayer Coating on Osseointegration of Endosteal Implants

**DOI:** 10.1021/acsbiomaterials.5c00249

**Published:** 2025-08-29

**Authors:** Vasudev Vivekanand Nayak, Justin E. Herbert, Bruno Luís Graciliano Silva, Sophie Kelly, Camila Suarez, Maria Castellon, Pawan Pathagamage, Estevam A. Bonfante, Lukasz Witek, Paulo G. Coelho

**Affiliations:** † Department of Biochemistry and Molecular Biology, 12235 University of Miami Miller School of Medicine, Miami, Florida 33136, United States; ‡ Dr. John T. Macdonald Foundation Biomedical Nanotechnology Institute (BioNIUM), University of Miami, Miami, Florida 33136, United States; § Biomaterials and Regenerative Biology Division, 5894 NYU College of Dentistry, New York, New York 10010, United States; ∥ Charles E. Schmidt College of Medicine, 306688 Florida Atlantic University, Boca Raton, Florida 33431, United States; ⊥ Trinity College of Arts and Sciences, 118712 Duke University, Durham, North Carolina 27708, United States; # Department of Oral Science and Translational Research, College of Dental Medicine, 2814 Nova Southeastern University, Fort Lauderdale, Florida 33314, United States; ∇ University of Miami Miller School of Medicine, Miami, Florida 33136, United States; ○ Department of Prosthodontics and Periodontology, Bauru School of Dentistry, 28133 University of São Paulo (USP), Bauru, SP 05508, Brazil; ◆ Department of Biomedical Engineering, NYU Tandon School of Engineering, Brooklyn, New York 11201, United States; ¶ Hansjörg Wyss Department of Plastic Surgery, NYU Grossman School of Medicine, New York, New York 10016, United States; †† Department of Oral and Maxillofacial Surgery, NYU College of Dentistry, New York, New York 10010, United States; ‡‡ DeWitt Daughtry Family Department of Surgery, Division of Plastic Surgery, University of Miami Miller School of Medicine, Miami, Florida 33136, United States; ⬡ Sylvester Comprehensive Cancer Center, University of Miami Miller School of Medicine, Miami, Florida 33136, United States

**Keywords:** modified triple salts, osseointegration, dental implants

## Abstract

**Background:** Improvements in osseointegration and bone healing as a result of surface modifications indicate that the time frame following implantation necessary to achieve biomechanical capacity for functional load-bearing may be reduced. In this context, a potassium peroxymonosulfate-based modified triple salt monolayer could potentially serve as a viable surface coating to further augment bone regenerative capabilities of endosteal implants. **Methods:** Implants with resorbable blast media textured surface [Tapered Pro 3DS RBT (Laser-Lok), BioHorizons] (CTRL) were treated with a potassium peroxymonosulfate-based modified triple salt coating process to generate a stabilized monolayer (Oxion). Prior to surgical intervention, implants were subjected to surface characterization. Subsequently, implants were evaluated in a large, preclinical sheep model (*n* = 14 sheep). A total of 12 implants were placed bilaterally in the submandibular ramus (3 implants per group per sheep per side) and allowed to heal for 3- and 12-weeks (7 sheep per time point). Following the allocated healing time, the animals were euthanized, mandibles harvested, and samples isolated for histomorphometric and nanoindentation analysis, along with biomechanical assessment through implant lateral load testing. **Results:** The Oxion coated implant's surfaces yielded lower contact angle (*p* < 0.001) and higher surface free energy values (*p* < 0.001) relative to the CTRL surface. Bone-to-Implant Contact (BIC) and Bone Area Fractional Occupancy (BAFO), which were used to quantify degrees of osseointegration, were statistically homogeneous at both healing times between Oxion and CTRL surfaces. Biomechanical testing, i.e. nanoindentation and lateral loading, demonstrated improved values for Oxion implants at both early and advanced healing time points compared to CTRL (*p* = 0.001). **Conclusion:** Implant failures continue to manifest during the initial months following implant insertion due to a variety of reasons, including inadequate osseointegration, or in cases involving clinical diseases and comorbidities. These findings suggest that the time frame following implantation necessary to achieve biomechanical capacity for functional load-bearing can be further reduced due to the Oxion surface coating in addition to the potential for enhanced early biomechanical integration relative to CTRL.

## Graphical abstract

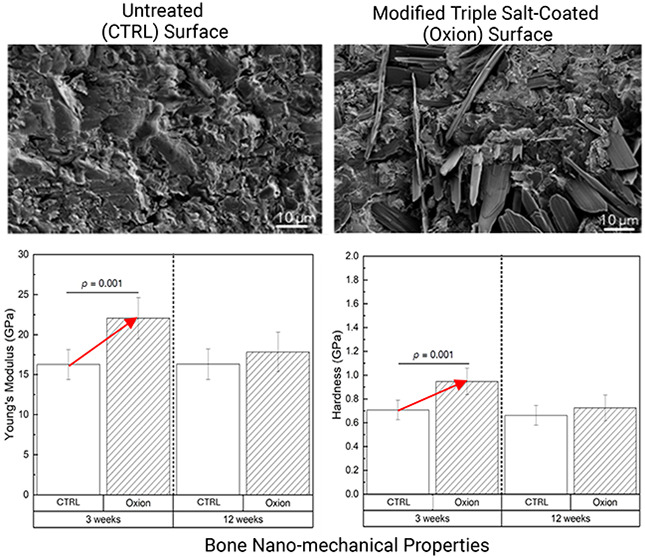

## 1 Introduction

Endosteal implants facilitate rehabilitation of partially or completely edentulous patients, and are considered the standard of care for oral rehabilitation.^1^ Unfortunately, implant failures continue to arise due to a variety of reasons, with the primary one being linked to inadequate osseointegration, occurring in ∼0.75–7.5% of patients during the initial months following implant insertion.^2–4^ Furthermore, osseointegration can be severely compromised in cases involving advanced clinical diseases/comorbidities, such as diabetes mellitus, osteoporosis, or osteoradionecrosis, etc.^5^ Such conditions present a substantial challenge in dental implantology, highlighting the need for improving endosteal implants to facilitate osseointegration after placement.

One aspect that can be modified is the implant’s surface, which is in direct contact with existing bone and has been demonstrated to be essential in accelerating early physiological loading of implants and minimizing crestal bone loss.^6^ Bone tissue in the mandible experiences physiological loading in addition to structural and functional adaptation to intrinsic biological factors.^7^ However, during implant placement, the implant swiftly occupies a large portion of the osteotomy, therefore reducing the gap between the osteotomy walls.^8^ In this scenario, the characteristics of the implant at varying length scales have been shown to influence bone healing, particularly when implant surfaces mimic the hierarchical architecture of native bone.^9–11^ In recent years biomedical research has shifted its focus from implant geometries to osseoinductive features of implant surfaces.^12,13^ Key surface attributes, including topography, wettability, and surface energy, have been demonstrated to significantly influence the osseointegration process.^14^

Osteogenic activity is essential in dictating osseointegration, particularly via contact osteogenesis.^15^ In this regard, enhancing the surface properties of the implant may promote bone tissue production and prevent the development of undesirable fibrous capsules—which can obstruct the stable integration of the implant surface with the peri-implant bone tissue.^16^ Consequently, a wide range of implant systems with diverse physicochemical surface characteristics have been developed to improve clinical outcomes.^17–25^ For example, laser micromachining of an implant collar to produce microscale channels, such as those seen in BioHorizons's (Birmingham, AL, USA) commercially available Laser-Lok implants, elicits greater clinical attachment levels and reduces peri-implant crestal bone loss relative to conventional, as-machined counterparts.^26,27^ These microchannels function as a biological seal promoting the adhesion of connective tissue and bone while preventing epithelial downgrowth.^28,29^ Similarly, in a clinical study evaluating laser ablation-modified implants at both the micro- and nano-length scales, a large amount of remodeled, osteonal bone was observed within the implant threads.^24^ As osteoblasts adhere more effectively to rougher implant surfaces, various other methods of surface modifications. such as high-pressure blasting with resorbable media and/or acid etching are gaining prominence.^30–33^ These modified surfaces have been shown to enhance osseointegration by augmenting the available surface area for cell adhesion, and have performed well in various preclinical and clinical cases with literature supporting their efficacy.^17–25^

In this context, recently, a potassium peroxymonosulfate (KHSO_5_)-based modified triple salt was introduced, which bonds to titanium oxide by acid deprotonation.^34^ The resulting monolayer was shown to be stable, resistant to change by γ radiation, and the potential to be incorporated as a monolayer bioactive surface coating on endosteal implants to further augment osseointegration.^34^ The current study aimed to build on these findings by evaluating the effect of the modified triple salt solution process on implant biocompatibility, bone regenerative and biomechanical capacities in a large translational (sheep) preclinical model.

## 2 Methods and Materials

### 2.1 Implant Surface Treatment and Characterization

Implants that were previously subjected to resorbable blast media texturing (RBT) and laser micromachining of the collar (Tapered Pro 3DS RBT (Laser-Lok), 4.2 mm × 9 mm, BioHorizons, Birmingham, AL, USA) were either left untreated (Control, CTRL) or subjected to a supplementary, patented, potassium peroxymonosulfate-based modified triple salt solution coating process (Experimental, Oxion) to form a stabilized monolayer on the surface.^34^ Briefly, the modified triple salt solution consisted of peroxymonosulfate (KHSO_5_) and potassium sulfate (K_2_SO_4_) in liquid format, and was converted to powder by lyophilization process. The obtained powder with approximate molar ratio 6:4 of persulfate to sulfate was dissolved in purified water to form reaction solution for monolayer coating.^34^ All implants used in this study were used directly as supplied by the manufacturer (BioHorizons, Birmingham, AL, USA).

Implant surfaces were imaged using a field emission scanning electron microscope (SEM) (Zeiss 300 FE-SEM, Oberkochen, Germany) at 5 kV and ∼100 pA and at a working distance of 8.0 mm. Surface roughness parameters: *R*_a_ (roughness average), *R*_q_ (root-mean-square average), *S*_a_ (arithmetical mean height), and *S*_q_ (root-mean-square height) were analyzed using built-in 3D profilometry software (Zeiss 300 FE-SEM, Oberkochen, Germany), with random areas (*n* = 3 per implant, 1 implant per group, ∼175 × 175 μm^2^ each) on the implant body chosen for the quantification of the surface roughness parameters. Additionally, elemental quantification using energy dispersive X-ray spectroscopy (EDS) was performed at random spots (*n* = 3 spots per implant, 1 implant per group, with a field with of ∼50 × 50 μm^2^ each) on the implant surfaces. Data acquisition for EDS was performed using a Quantax 200 XFlash 6160 detector (Bruker, Billerica, MA, USA) integrated with the SEM (Zeiss 300 FE-SEM, Oberkochen, Germany) and normalized as described elsewhere.^35^ Elements that accounted for <5% (atomic %) of the total surface composition were considered to be trace. All measurements were conducted under high vacuum conditions at 5 kV, ∼100pA, and using an electron beam aperture of 60 μm. The working distance was maintained at 8.0 mm, which corresponds to the analytical working distance for this equipment configuration. For all SEM and EDS tests, implants were imaged without additional sputter coating.

To measure surface energy and contact angle, grade 5 Titanium disks (10 mm in diameter and 1 mm in height) were subjected to the same surface treatments as described above, and were used directly as supplied by the manufacturer (BioHorizons, Birmingham, AL, USA). All disks (*n* = 9 disks per group) were horizontally positioned on a contact angle goniometer (OCA 30, Data Physics Instruments GmbH, Filderstadt, Germany), following which contact angle and surface free energy were determined using a pre-established protocol.^36^ In brief, 500 μL droplets each of distilled water, ethylene glycol, and diiodomethane were sequentially deposited on the surface of each disk using the automated dispensing system of the goniometer to record contact angles. Subsequently, the Owens-Wendt-Rabel-Kaelble (OWRK) method was utilized to calculate surface energy.^37^

### 2.2 Surgical Procedure

The sample size for this investigation was established *a priori* by power analysis, which suggested that at least 14 implants per group per time point were necessary to attain 80% power at an α level of 0.05 and an effect size of 0.45–0.5. The sheep mandibular model permitted nesting of additional implants within each animal (due to the large size of the mandible). Therefore, a total of 12 implants were placed bilaterally in the submandibular ramus of each sheep (3 implants per group per side) as per schematic shown in Figure 1. Upon approval from École Nationale Vétérinaire d’Alfort (Maisons-Alfort, Ile-de-France, France) Institutional Animal Care and Use Committee (file and notice numbers: 13–011 and 05/14/13–3, respectively), adult sheep (*n* = 14) were obtained and allowed to acclimate for approximately 1 week at the animal facility. All surgical interventions were carried out in a sterile setting and under general anesthesia. The sheep were administered sodium pentothal (15–20 mg/kg) in Normasol solution by injection into the jugular vein. Anesthesia was maintained with isoflurane (1.5–3%) in O_2_/N_2_O (50/50). Vital signs were monitored using ECG, SpO_2_, and final tidal CO_2_. Prior to incision, the surgical site was shaved and disinfected with an iodine solution. Mandibular bone exposure was performed bilaterally, and six osteotomies were created in each submandibular region (left and right, respectively) using rotary instrumentation under continuous irrigation, as per the implant manufacturer’s recommended drilling sequence.

**Figure 1:**
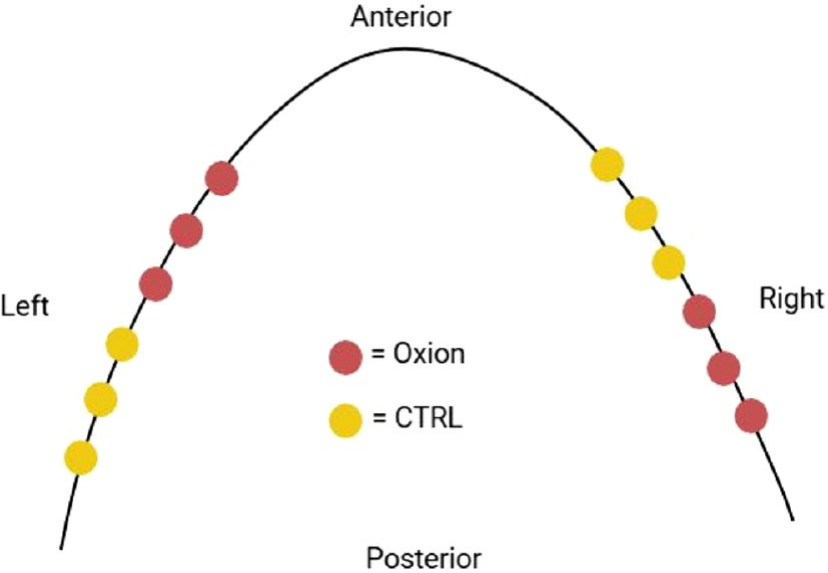
Schematic representation of the implants placed in the mandibular bone. Created on Biorender.com.

Implants were positioned in an interpolated manner. Cefazolin (500 mg) was administered preoperatively and postoperatively via intravenous injection to reduce complications, and animals were provided food, water *ad libitum*. Previous research demonstrated osteoid tissue presence within the healing chambers after 3 weeks in vivo, indicative of initial bone formation.^6^ This was accompanied by mature osteonic structures and evidence of revascularization through intramembranous-like healing pathways at 12 weeks.^6^ Thus, the present investigation designated postsurgical intervention intervals of 3- and 12- weeks to signify these healing periods. In brief, 3- and 12- weeks after surgical intervention, animals (*n* = 7 per time point) were euthanized according to the approved protocol and samples were harvested *en bloc*. *N* = 14 samples per group per time point were used for each of the following analyses: histomorphometric analysis, biomechanical testing, and nanoindentation testing.

### 2.3 Lateral Load Testing

Early physiological loading after implant placement can negatively affect the supporting bone structure and compromise healing outcomes. It has been observed that the lateral load component of masticatory forces may have a more detrimental effect on cortical bone yielding and bone loss compared to axial or vertical loading.^38,39^ As such, samples were adapted to an appropriate implant removal tool/key (Figure 2a), and constrained on a universal testing machine (Instron, Norwood, MA, USA) equipped with a ± 1000 N load cell. Samples were subjected to lateral loading at a rate of 1 mm/min with the implant oriented such that loading would enable interfacial fracture between bone and the healing chambers (Figure 2a.1). Samples were excluded if grip slippage or preloading fracture was detected by large unintended loads/displacements or appreciable discontinuities, respectively, in the test data. The maximum lateral load (*N*) and the area under the load–displacement curve (work-to-maximum load, *J*) was recorded for each implant (Figure 2b). This test routine was adapted from a previously established procedure to evaluate implant biomechanical parameters due to lateral loading.^40,41^

**Figure 2:**
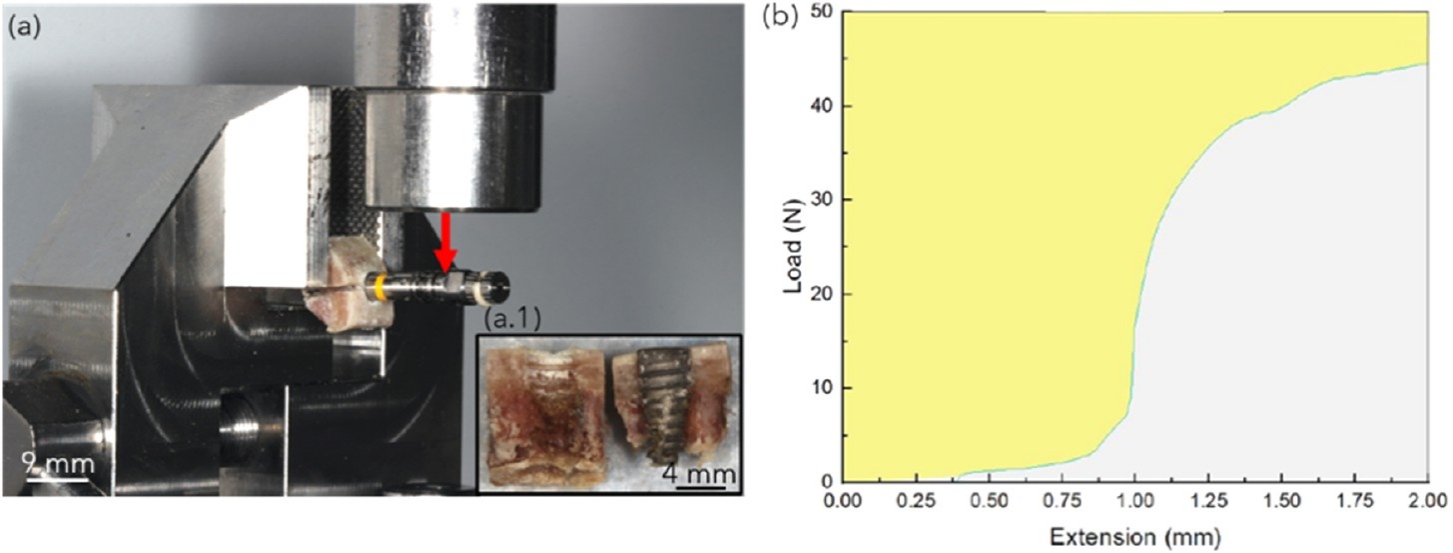
(a) Lateral loading test setup showing the implant in bone positioned in the test jig and a load applied in the direction of the arrow (in red) against the implant removal tool/key, (a.1) representative sample pictured at the end of the test, showcasing interfacial fracture between the implant surface and bone, (b) representative load vs extension/displacement curve showing the area under the curve (in gray).

### 2.4 Histological Processing and Histomorphometric Analysis

The samples underwent sequential dehydration in 70 to 100% ethanol (EtOH). Following the dehydration process, samples were submerged in methyl salicylate followed by infiltration and embedding in a methacrylate-based resin. The embedded blocks were sliced into thin sections of ∼300 μm using a low-speed precision wafering saw (Isomet 2000, Buehler Ltd., Lake Bluff, IL, USA). Each slice was then glued to acrylic slides using a cyanoacrylate-based adhesive (Loctite 408, Henkel AG & Co. KGaA, Düsseldorf, Germany) and subsequently reduced to a thickness of ∼90 μm (±10 μm) on a grinding machine (Metaserv 3000, Buehler, Lake Bluff, IL, USA) equipped with a series of SiC abrasive papers (400, 600, 800, and 1200 grit, respectively) under copious irrigation with water. Slides were then polished using a microfiber cloth coated with a polishing solution (MicroPolish Alumina (1 μm), Buehler, Lake Bluff, IL, USA) for 1 min and rinsed thoroughly with water. Subsequently, the slides were stained with Stevenel’s blue and Van Gieson picro-fuchsin (SVG), as shown in the literature.^42^ Digital scanning of the stained sections was performed on an automated slide scanning system (Aperio CS2, Leica, Wetzlar, Germany).

Bone-to-Implant Contact (BIC) was determined by calculating the ratio of the perimeter of the implant surface in direct contact with new bone (bone perimeter) to the perimeter of the entire implant (implant perimeter), expressed as a percentage. Bone Area Fractional Occupancy (BAFO) was determined by the ratio of the bone area within the threads (bone area) to the total area of the threads (thread area), expressed as a percentage. Both histomorphometric parameters, illustrated in Figure 3 were quantified, as demonstrated previously, using ImageJ (National Institutes of Health, Bethesda, MD).^43^ BIC and BAFO are quantitative metrics of osseointegration and serve as objective measures of bone healing.

**Figure 3:**
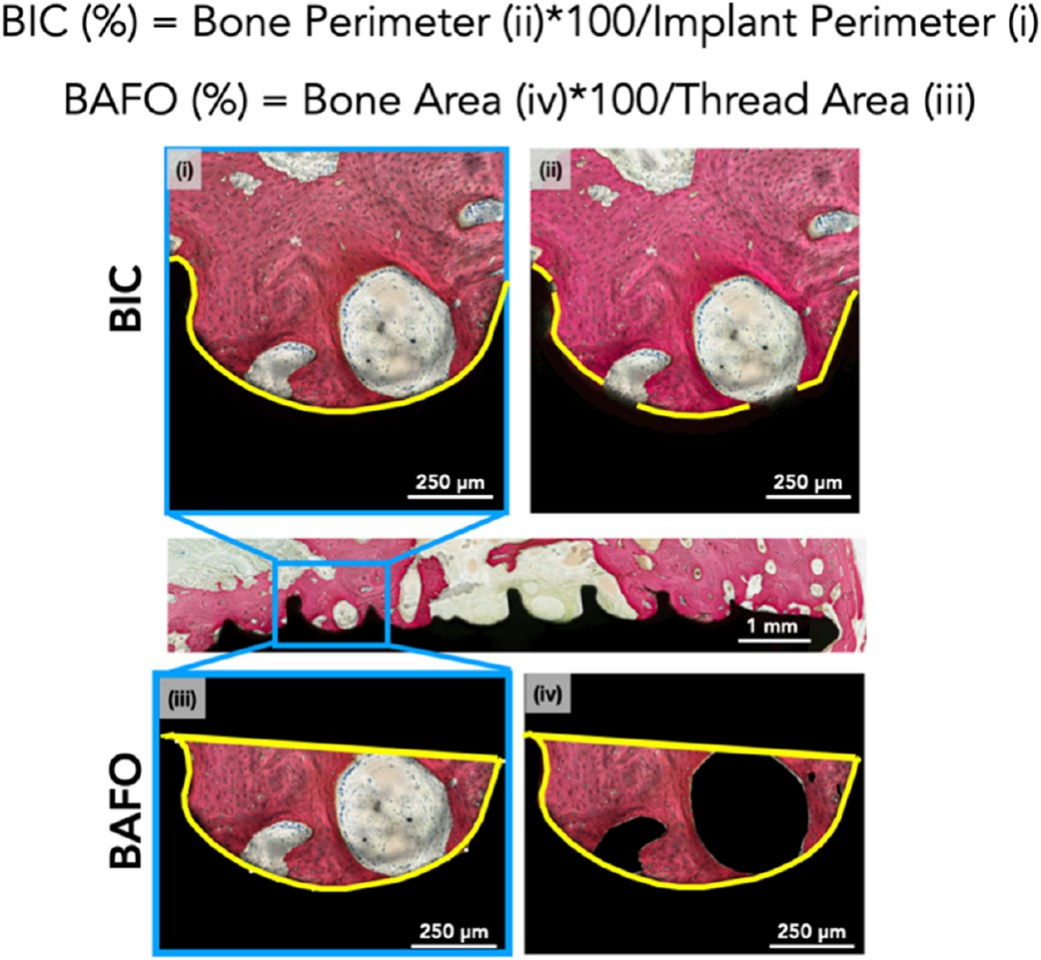
Representative histomicrograph showing the threaded region of the implant (one side shown), with bone in red. Inserts depict the region of interest (highlighted in yellow, specifically focusing on one of the healing chambers for ease of representation).

### 2.5 Nanoindentation Testing

To accurately ascertain the influence of surface characteristics on bone–implant integration, evaluation of localized biomechanical parameters (in proximity to the bone-implant interface) through nanoindentation was necessitated. Nanoindentation tests were performed within the healing chambers and in proximity to the bone-implant interface as illustrated in Figure 4. The histological slides for nanoindentation testing were processed in a similar fashion as above (Section 2.4). Following the alumina-based suspension, a series of progressively finer diamond-based suspensions (9 to 0.1 μm, Electron Microscopy Sciences, Hatfield, PA, USA) were used to further polish the slides on a microfiber cloth and ultrasonicated for 3 min. For each implant (1 slide per implant), nine indentations spaced 10 μm in the *x*- and *y-*axis (in a 3 × 3 grid for a total area of ∼20 × 20 μm^2^) were performed on bone within a single region of interest within the healing chamber (Figure 4.i—dashed blue box). Indentations were performed with a Berkovich tip (Hysitron TI 950 Nanoindenter, Bruker, Billerica, MA, USA) at a rate of 60 μN/s to a peak load of 300 μN.^44^ All load–displacement indentation curve were checked for potential issues such as those arising from contact or large unintended displacements. For each indentation, both the reduced modulus (*E*_r_, in GPa) and the hardness (*H*, in GPa) were computed. Subsequently, Hertzian contact mechanics were applied to transform the reduced modulus (*E*_r_) to Young’s modulus (*E*, GPa) using the relationship^45^

$$E=\frac{\left(1-v_{b}^{2}\right)E_{i}E_{r}}{E_{i}-\left(1-v_{i}^{2}\right)E_{r}}$$

Where, *v*_b_ is the Poisson’s ratio of bone, *E*_i_ is the elastic modulus of the indenter, and *v*_i_ is the Poisson’s ratio of the indenter.

**Figure 4:**
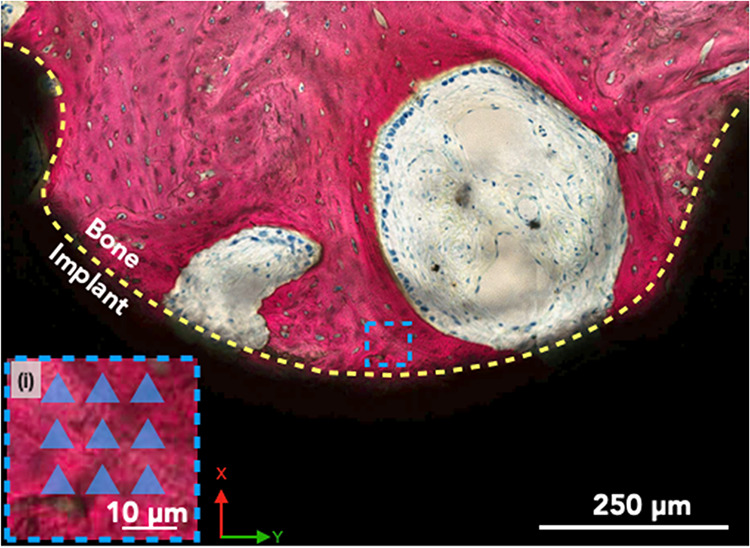
Representative histological section of the bone (in red) and implant (in black), with the dashed yellow curve depicting the bone-implant interfacial region within the healing chamber. The dashed blue square represents the location of the indents in proximity to the bone-implant interface—magnified in insert (i). (i) The blue triangles are schematic representations (not to scale) of the 9 indents spaced 10 μm in the *x*- and *y*-axis, in a 3 × 3 grid for a total area of ∼20 × 20 μm^2^ in the proximity of the bone implant interface within the healing chamber of the implant macrogeometry.

### 2.6 Statistical Analysis

Statistical evaluation of all outcome variables was performed using mixed model analysis of variance with fixed factors of time and group. All statistical analyses of data were performed using IBM SPSS (v29, IBM Corp., Armonk, NY). All values were reported as mean ± 95% confidence interval (95% CI), with *p* < 0.05 indicating significance, unless otherwise specified.

## 3 Results

### 3.1 Implant Surface Characterization

Scanning electron microscopy showed that the CTRL group had a smoother, uniform surface (Figure 5a,b), while the Oxion group displayed evenly distributed needle-like structures on the implant surface (threads and healing chambers) (Figure 5c,d). The Oxion group also presented higher mean roughness values versus CTRL: *R*_a_ (0.326 μm vs 0.130 μm), *R*_q_ (0.401 μm vs 0.164 μm), *S*_a_ (0.246 μm vs 0.098 μm), and *S*_q_ (0.312 μm vs 0.124 μm). The surface chemistry assessment of the CTRL group primarily showed the presence of Titanium (Ti; 50.94%), and Vanadium (V, 33.69%), with trace levels of Oxygen (O, 4.79%), Aluminum (Al, 4.74%), Nitrogen (N, 3.05%), Carbon (C, 2.05%), and Phosphorus (P, 0.8%). Relative to the CTRL group, the Oxion-treated surface presented elevated levels of Potassium (K, 13.59%) and Sulfur (S, 8.23%). Of note, trace elements like P and C were not detected on the modified triple salt-treated implant surfaces. This could be due to the presence of these potassium peroxymonosulfate crystals that are deposited upon a grit-blasted surface, possibly hindering the quantification of the underlying trace elements as a result of the low EDS detection depth. The Oxion surface presented with a significantly lower contact angle (*p* < 0.001, Figure 6a), and increased both polar and dispersive components of surface energy compared to CTRL (*p* < 0.001, Figure 6b).

**Figure 5:**
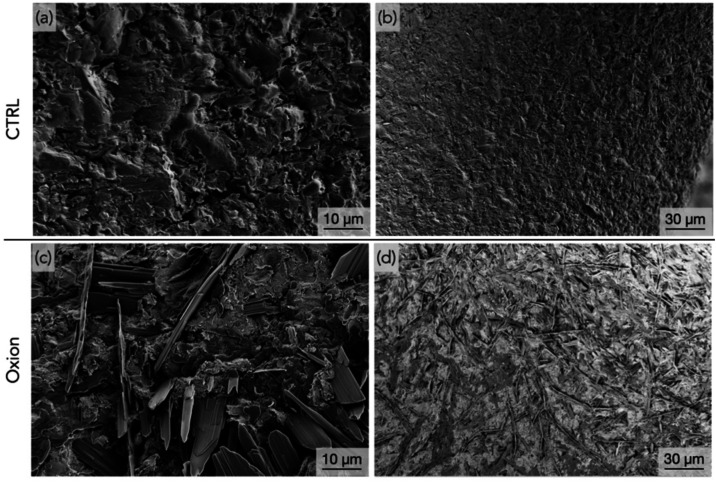
Representative high and low magnification scanning electron micrographs of the (a, b) CTRL (TP 3DS surface presented in previous work^46^), and (c, d) Oxion surfaces, respectively.

**Figure 6:**
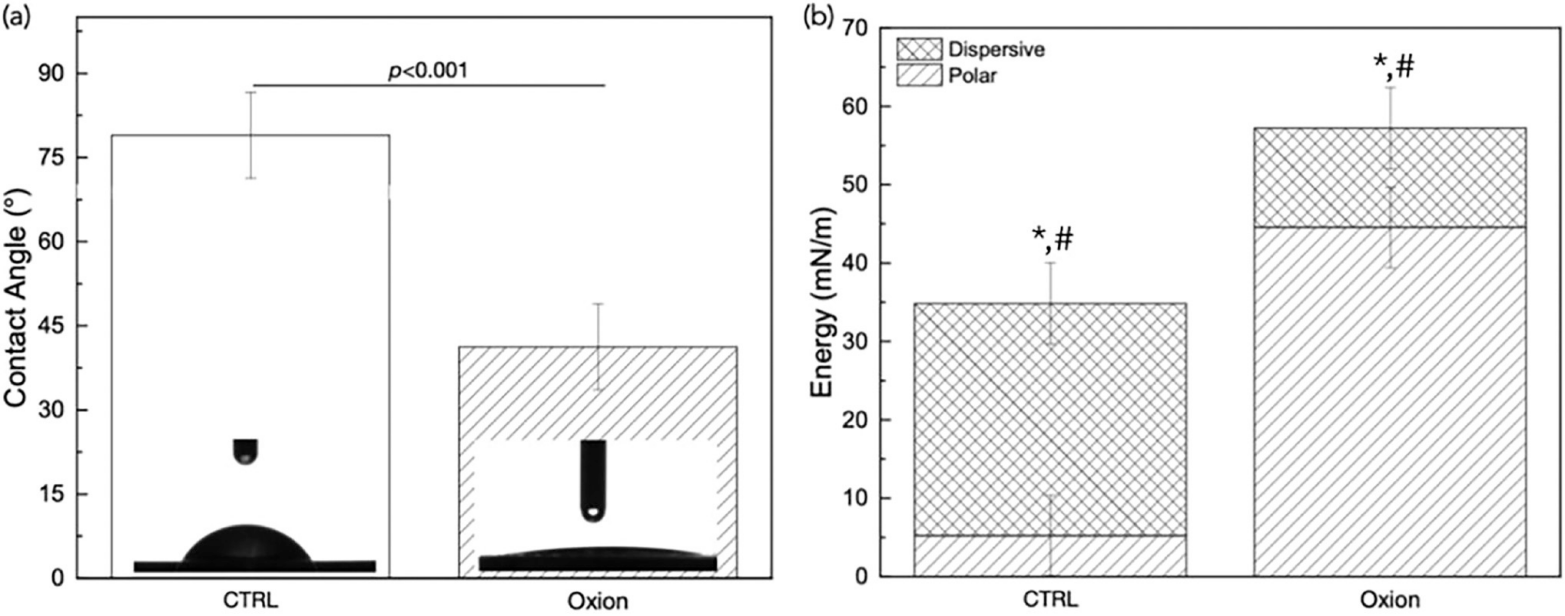
(a) Contact angle values (in degrees), with inserts showing a visual representation of a probe liquid (water), and (b) surface energy measurements (in mN/m) presented as a stacked column plot of the polar and dispersive components. Data presented as means and corresponding 95% confidence intervals with *p* < 0.05 being statistically significant. Symbols (* and #) indicate statistical significance in polar and dispersive components of surface energy, respectively, between CTRL and Oxion groups (*p* < 0.001).

### 3.2 Lateral Load Testing

No significant differences were observed in both lateral load and area (work-to-maximum load) data between the CTRL and Oxion surface at the early stages of osseointegration (3 weeks). However, at 12 weeks, the Oxion surface resulted in higher lateral loads (*p* = 0.001) and therefore a significantly greater area under the load–displacement curve (*p* = 0.001) (Figure 7a,b). Of note, between 3- and 12-weeks, the CTRL surface did not elicit a higher load to failure or area under the curve. Contrarily, the Oxion surface presented significantly greater values of maximum lateral load at failure (*p* = 0.010) and work-to-maximum load (*p* = 0.025) at 12 weeks relative to the early healing time point (Figure 7c,d).

**Figure 7:**
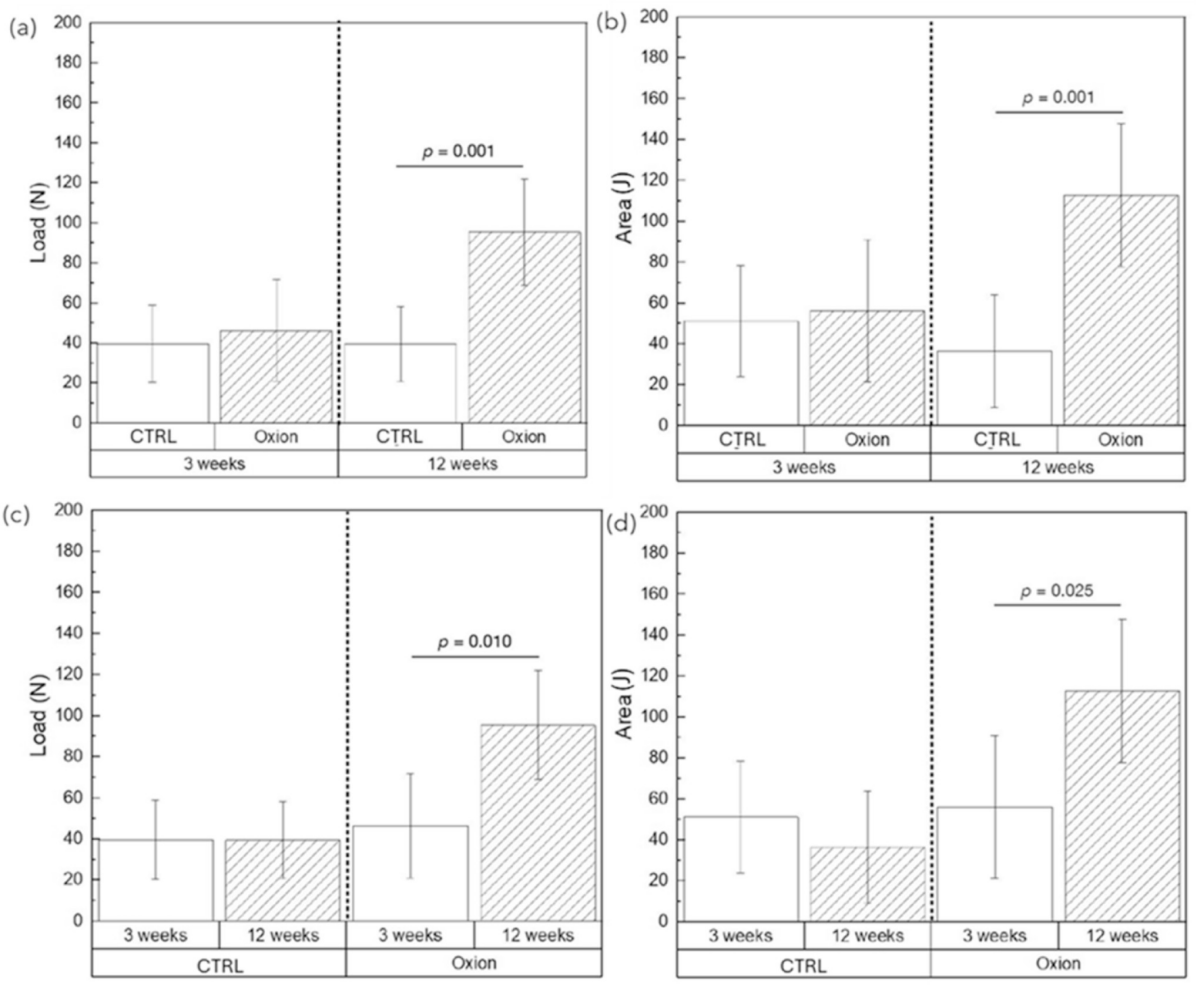
Load (*N*) and area (work-to-maximum load, *J*) data (a, b) compared as a function of groups at individual time points, and (c, d) compared as a function of time point within an implant group (CTRL or Oxion). Data presented as means and corresponding 95% confidence intervals. *p* < 0.05 is statistically significant.

### 3.3 Histological and Histomorphometric Findings

Quantitative evaluation of BIC and BAFO showed no significant differences between the CTRL and Oxion groups at either time point (Figure 8a,b). However, a significant increase in both outcome variables was observed in the CTRL (BIC and BAFO: *p* = 0.001) and Oxion (BIC: *p* = 0.002; BAFO: *p* = 0.010) groups between 3- and 12-weeks (Figure 8c,d). Qualitative analysis of the histological micrographs revealed successful osseointegration of implants irrespective of surface treatment at both time points (Figure 9, and Supporting Figures S1–S2). At 3 weeks, the implant crestal modules (cervical area) where initial interlocking took place was surrounded by woven bone in both the CTRL (Figure 10a,b) and Oxion groups (Figure 10c,d). At the advanced healing time point, bone growth appeared appositional with evidence of remodeling sites within the healing chambers, irrespective of the implant group (Figure 10e–h).

**Figure 8:**
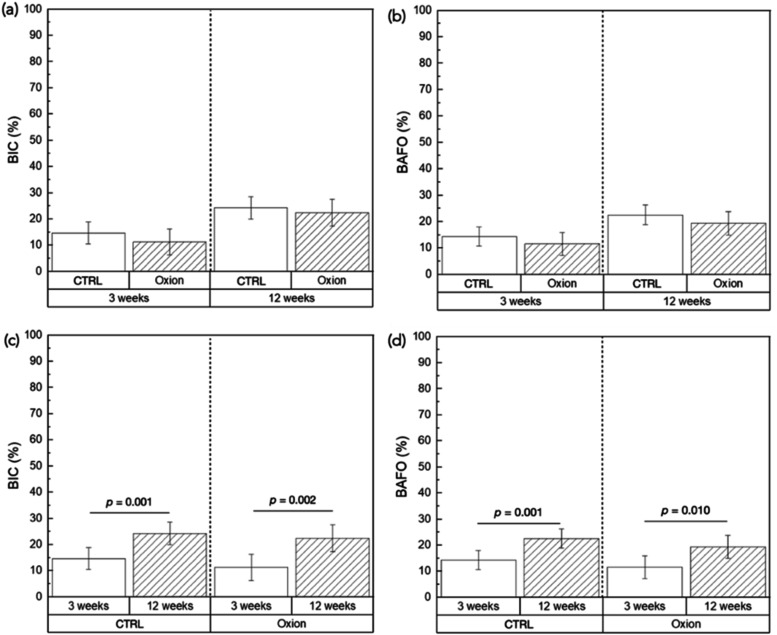
BIC (%) and BAFO (%) data (a, b) compared as a function of groups at individual time points, and (c, d) compared as a function of time point within an implant group. Data presented as means and corresponding 95% confidence intervals. *p* < 0.05 is statistically significant.

**Figure 9:**
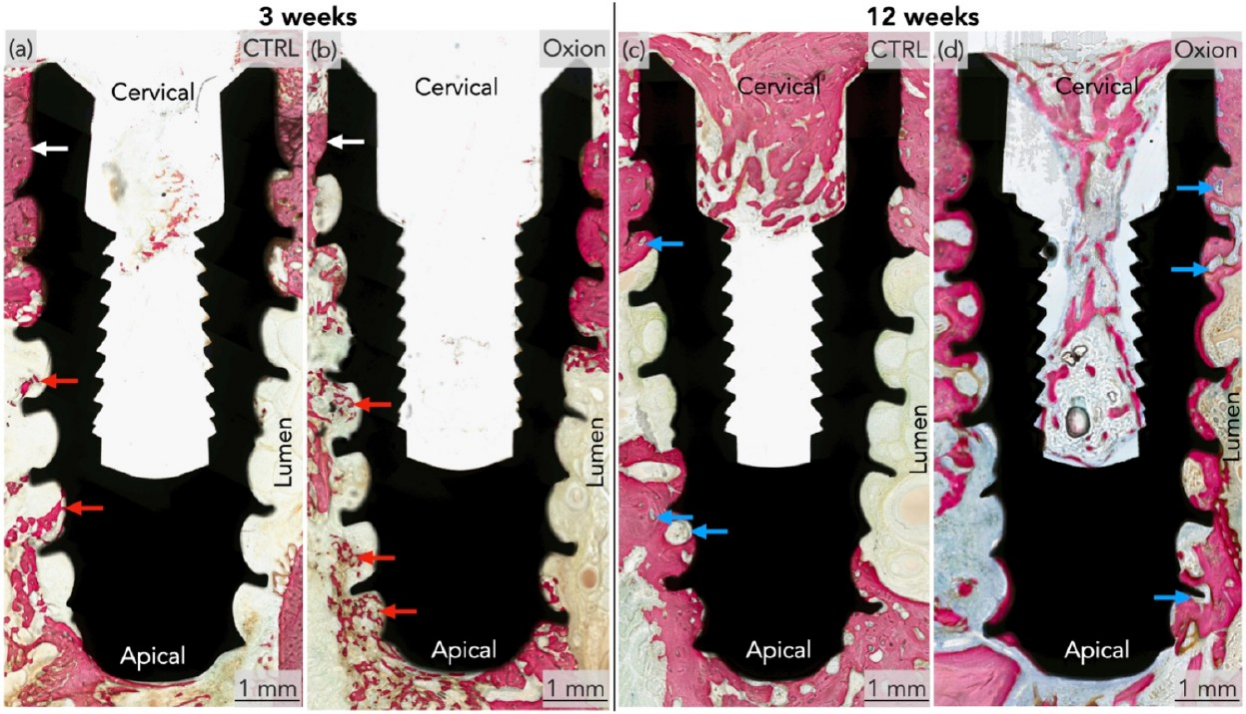
Representative low magnification histological overviews of the CTRL and Oxion groups at (a, b) 3- and (c, d) 12-weeks, respectively, showing calcified tissue in red and implant in black. White arrows denote bone growth at the implant crest module in both groups at the 3-week time point, whereas red arrows indicate de novo bone formation at 3 weeks in the healing chambers in proximity to the lumen. Blue arrows highlight bone remodeling sites in both groups at 12 week. Cervical = near the implant collar, and apical = in proximity to the cutting edge of the implant macro-geometry.

**Figure 10:**
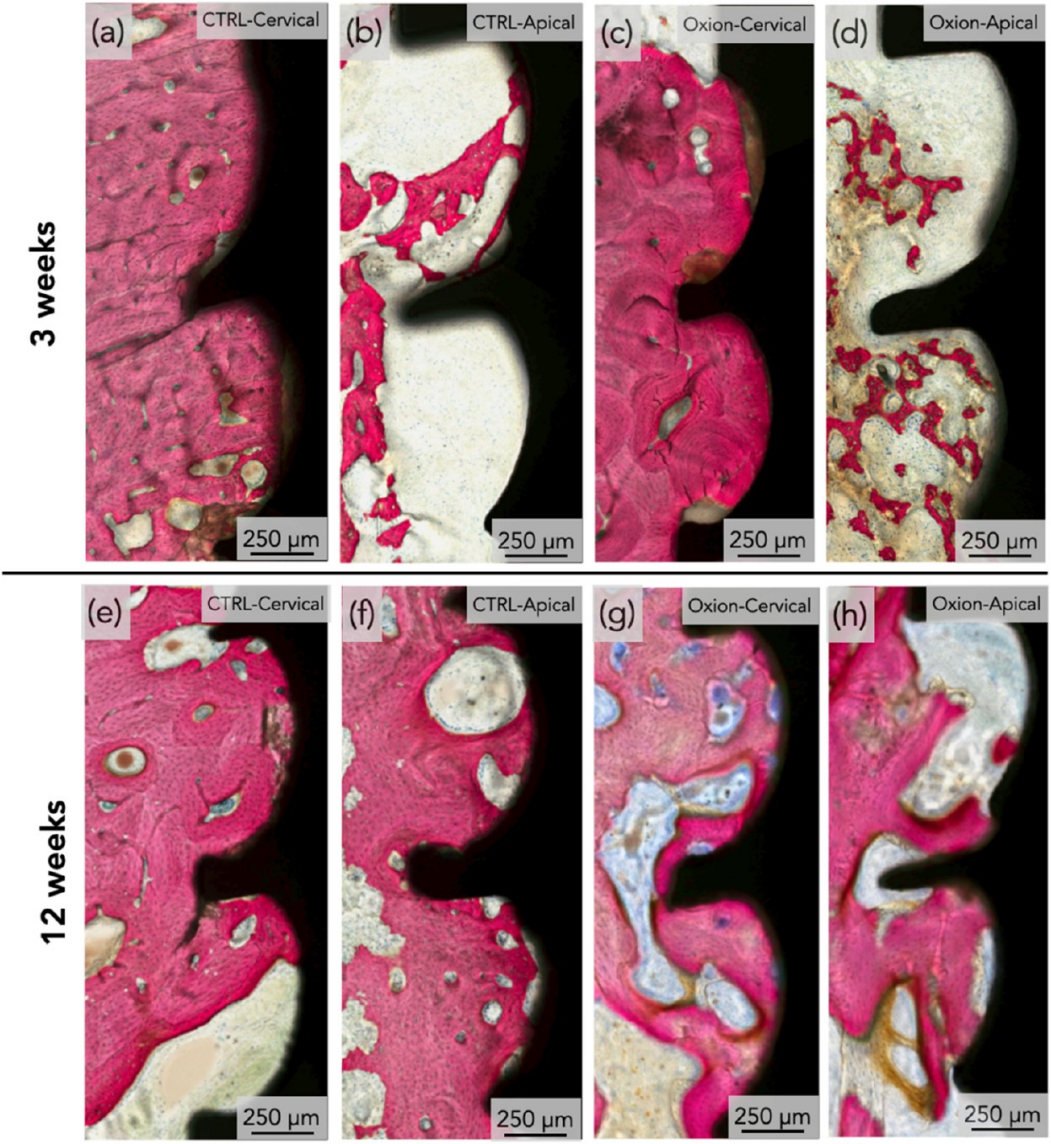
Representative high magnification histomicrographs of the: (a, b) CTRL group at 3 weeks presenting the cervical and apical aspects, respectively; (c, d) Oxion group at 3 weeks presenting the cervical and apical aspects, respectively. Representative high magnification histomicrographs of the: (e, f) CTRL group at 12 weeks presenting the cervical and apical aspects, respectively; (g, h) Oxion group at 12 weeks presenting the cervical and apical aspects, respectively. Calcified tissue is presented in red and implant in black. Cervical = near the implant collar, and apical = in proximity to the cutting edge of the implant macro-geometry.

### 3.4 Nanoindentation Testing

Oxion coated implants in the mandible demonstrated a significant increase in Young’s Modulus (*E*) and Hardness (*H*) at the early healing time point (3 weeks) compared to the CTRL surface (*p* = 0.001) (Figure 11a,b). No differences in either parameter was observed between the two groups at the advanced healing time point (12 weeks) (Figure 11a,b). Of note, a significant decrease in both outcome variables was recorded in the Oxion group (*E*: *p* = 0.022, *H*: *p* = 0.006) between 3- and 12-weeks (Figure 11c,d).

**Figure 11:**
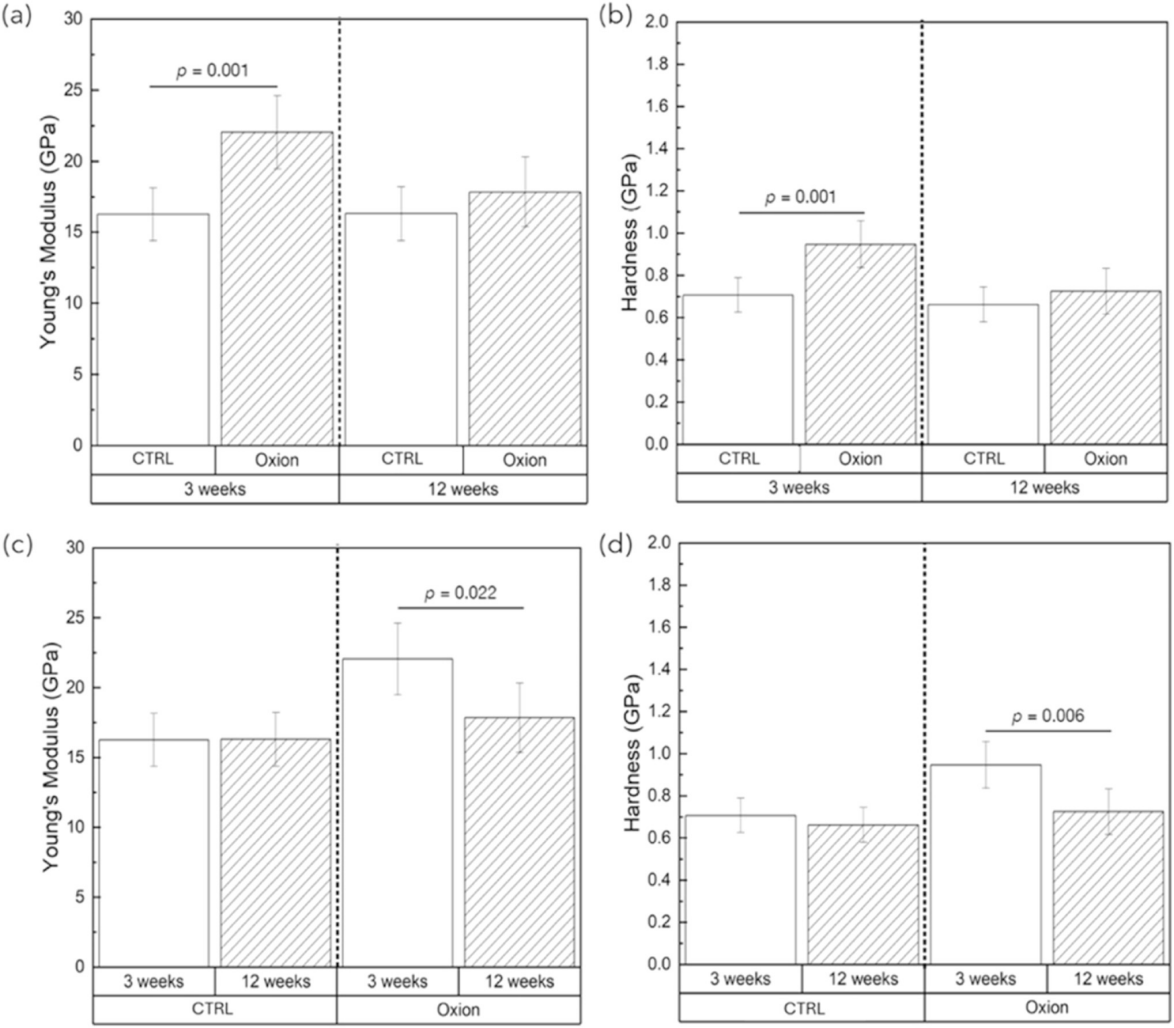
Young’s modulus (GPa) and Hardness (GPa) data (a, b) compared as a function of groups at individual time points, and (c, d) compared as a function of time point within an implant group. Data presented as means and corresponding 95% confidence intervals. *p* < 0.05 is statistically significant.

## 4 Discussion

The composition of the modified triple salt monolayer coating was potassium peroxymonosulfate, not as a standalone molecule, which was covalently and/or ionically bound to the implant.^34^ Potassium peroxymonosulfate (shown in literature and corroborated with EDS/elemental analysis in the current study) resulted in the modified triple salt proxy functional group of K^+^[HSO_5_]^−^.^34^ The Oxion surface presented a significant increase in surface wettability/surface energy compared to CTRL. Trends in the literature suggest that an increase in surface energy and surface wettability can regulate bone healing around implant surfaces.^47,48^ Furthermore, hydrophilic surfaces support homogeneous spatial osteoblastic cell growth, mineral deposition, and increase osteoblastic cell mineralization, compared to hydrophobic surfaces.^47^ It is important to note that the surface energy is dependent on the type of bonds. These bonds can be divided into primary (covalent and/or ionic) or secondary type (typically van der Waals)—the former of which contributes predominantly toward the polar component of surface energy,^49^ as seen in the Oxion group.

BIC and BAFO outcomes were statistically comparable for CTRL and Oxion surfaces at 3 and 12 weeks, though mean values trended higher in the CTRL group. Notably, no adverse effects on bone healing or systemic response were observed via histological analysis, specifically within the time periods evaluated in this study, suggesting that the Oxion surface did not compromise early bone formation processes. Oxidation of the implant surface, albeit through techniques different from the one used in the current study, has been shown to effectively modify the surface composition to elicit enhanced bone regenerative outcomes.^50^ For example, the osteoconductive capabilities of anodized implant surfaces facilitated rapid integration with bone in a ten-year follow-up of immediately loaded implants featuring anodized surfaces, indicating a survival rate of ∼98%.^50,51^ Another randomized clinical investigation showed that anodized implant survival rates (∼95%) surpassed those of as-machined counterparts (∼85%).^50,52^

From a perspective of biomechanical competence, bone-implant integration and bone regeneration in areas away from the implant surface necessitate distinct assessments. The development of new bone in regions in proximity to- and away from- the implant surface may engage distinct osteogenic mechanisms, both of which are crucial to the biomechanical efficacy of the load-bearing implant.^53–56^ Bone repair in areas away from the bone-implant interface region not only involves *de novo* formation and enhanced wound healing but necessitates remodeling of surrounding tissue owing to the stresses induced by implant placement and subsequent physiological loading. Higher biomechanical competence (lateral load and work-to-maximum load) at 12 weeks in the Oxion group could be due to the presence of the monolayer coating. However, it is important to note that mean values of BIC and BAFO trended higher in the CTRL group relative to Oxion treated counterparts (although no significant differences were observed). The exact reason for this discrepancy between the histomorphometric findings and biomechanical test data is not fully understood. However, if the examination of bone biomechanical competence encompasses an excessive volume of adjacent tissues, the effect of the implant surface modification on biomechanical findings may be diminished especially at the shorter healing time points immediately following surgical implantation.

To accurately ascertain the influence of surface characteristics on bone–implant integration at the initial phase of bone formation, evaluation of localized biomechanical parameters through nanoindentation was necessitated. The increase in bone elastic modulus and hardness relative to untreated implants could be due to enhanced hybrid (interfacial and intramembranous-like) healing around the surface at the early healing time point.^57,58^ Healing chambers filled with the blood clot have been described to evolve toward osteogenic tissue that subsequently ossify through this hybrid pathway.^6^ Specifically, the blood filling the space between bone and the ‘bioactive’ implant surface can rapidly develop toward intramembranous ossified connective tissue networks shortly following implant placement.^6^ The subsequent reduction in Young’s modulus and hardness of bone at 12 weeks compared to the 3-week time point in the Oxion group can potentially be attributed to advanced stages of bone-implant interfacial remodeling. To elaborate, remodeling at this stage in the healing cascade typically involves continual removal and replacement of woven bone with proteinaceous matrix, and subsequent mineralization of the matrix to form new bone, and as a result, the resorption of previous rapidly ossified woven bone could be responsible for the reduction in *E* and *H* to normal physiological levels at 12 weeks, where the remodeling phase is nearing completion.^59–61^

From a clinical perspective, these improvements at the early healing time point could facilitate rapid physiological loading in cases involving fixed hybrid technologies, or single implant restorations (for example, in the anterior maxilla). More importantly, based on the histological trends and early biomechanics in the literature pertaining to bioactive surfaces,^44,62–65^ the Oxion surface could benefit patients presenting with systemic factors (such as diabetics, osteoporosis, and/or metabolic syndrome that are known to compromise bone regeneration) by supporting crestal bone healing and maintenance—key aspects to short- and long-term implant success. However, these trends are not confirmed by the outcomes presented in the current study and warrant follow-up evaluation in compromised, preclinical wound healing models.

This study examined a surface modification that has not been evaluated in the existing literature, particularly in large translational models, and aimed to establish a baseline for future preclinical/clinical research.^34^ The 12-week end point and sample size restricted the ability to discern long-term performance differences between groups, therefore upcoming efforts should focus on determining the specific biological factors that influence bone healing at time intervals >12 weeks in the presence of these surface modifications in larger cohorts. This necessitates follow-up evaluation on specific biological markers of inflammation and bone remodeling; robust surface characterization via transmission electron microscopy, X-ray photoelectron spectroscopy, high-resolution synchrotron radiation microtomography; in addition to an evaluation of the dissolution/degradation mechanisms of the Oxion surface. Moreover, the process parameters conventionally used to synthesize bioactive implant surfaces via electrochemical techniques like anodic oxidation, or via plasma spraying differ from the modified triple salt coating process utilized in the current study.^34,66–68^ This requires a direct comparison of the Oxion surface to other commercially available bioactive implant surfaces to fully understand the benefits of the modified triple salt monolayer on bone formation and healing for clinical translation. On the other hand, location of implant placement within the mandible has been shown to affect osseointegration outcomes and implant success rates resulting from changes in vascularity and bone density.^69,70^ For example, a previous clinical study evaluated bone density at various dental implant sites, where findings revealed higher mean bone density in the anterior region of the mandible relative to the posterior aspect.^70^ This factor was not accounted for in the analysis of outcome variables in current study necessitating follow-up evaluation.

While the results validate the benefits of the Oxion surface, it has been established that osseointegration is also directly influenced by hierarchical implant hardware design.^6^ Thus, an implant macro-geometry that allows the formation of healing chambers would be better-suited to improve the bone regenerative potential at the micro- and nano-meter length scales, and more importantly, to further hasten early osseointegration followed by improved long-term implant survival rates. Therefore, the translation of the Oxion surface while also considering implant macro-geometric parameters remains to be explored.

## 5 Conclusions

Within the limitations of the study, the findings suggest that the required preloading phase may be decreased owing to the Oxion surface in addition to potential enhanced early biomechanical integration/osseointegration relative to the CTRL surface.

## Supplementary Material



## Notes

Additional histomicrographs for the experimental groups (Oxion and CTRL surface) at 3-week (Supporting Figure S1), and 12-week (Supporting Figure S2) healing times (PDF)
