# Supplementary material for: Impact of Modified Triple Salt Monolayer Coating on Osseointegration of Endosteal Implants
Source: ACS Biomater Sci Eng. 2025 Aug 29;11(10):5850–61. doi: 10.1021/acsbiomaterials.5c00249 (PMC12522098; doi:10.1021/acsbiomaterials.5c00249)
Supplement: Supplementary file 1 [file ab5c00249_si_001.pdf]

Supporting Information for Publication

Impact of Modified Triple Salt Monolayer Coating on Osseointegration of Endosteal Implants

Vasudev Vivekanand Nayak[1,2], Justin E. Herbert[1], Bruno Luís Graciliano Silva[3], Sophie Kelly[4], Camila Suarez[5], Maria Castellon[6], Pawan Pathagamage[7], Estevam A. Bonfante[8], Lukasz Witek[3,9,10,11]*, Paulo G. Coelho[1,2,12,13]

[1] Department of Biochemistry and Molecular Biology, University of Miami Miller School of Medicine, Miami, FL, USA

[2] Dr. John T. Macdonald Foundation Biomedical Nanotechnology Institute (BioNIUM), University of Miami, Miami, FL, USA

[3] Biomaterials and Regenerative Biology Division, NYU College of Dentistry, New York, NY, USA

[4] Charles E. Schmidt College of Medicine, Florida Atlantic University, Boca Raton, FL, USA

[5] Trinity College of Arts and Sciences, Duke University, Durham, NC, USA

[6] Department of Oral Science and Translational Research, College of Dental Medicine, Nova Southeastern University, Fort Lauderdale, FL, USA

[7] University of Miami Miller School of Medicine, Miami, FL, USA

[8] Department of Prosthodontics and Periodontology, Bauru School of Dentistry, University of São Paulo (USP), Bauru, SP, Brazil

[9] Department of Biomedical Engineering, NYU Tandon School of Engineering, Brooklyn, NY, USA

[10] Hansjörg Wyss Department of Plastic Surgery, NYU Grossman School of Medicine, New York, NY, USA

[11] Department of Oral and Maxillofacial Surgery, NYU College of Dentistry, New York, NY, USA

[12] DeWitt Daughtry Family Department of Surgery, Division of Plastic Surgery, University of Miami Miller School of Medicine, Miami, FL, USA

[13] Sylvester Comprehensive Cancer Center, University of Miami Miller School of Medicine, Miami, FL, USA

***Corresponding Author:** Lukasz Witek MSci, PhD; address: 345 E. 24th St., Room 806, New York, NY 10010 USA; phone: 212 998 9269; email: lukasz.witek@nyu.edu

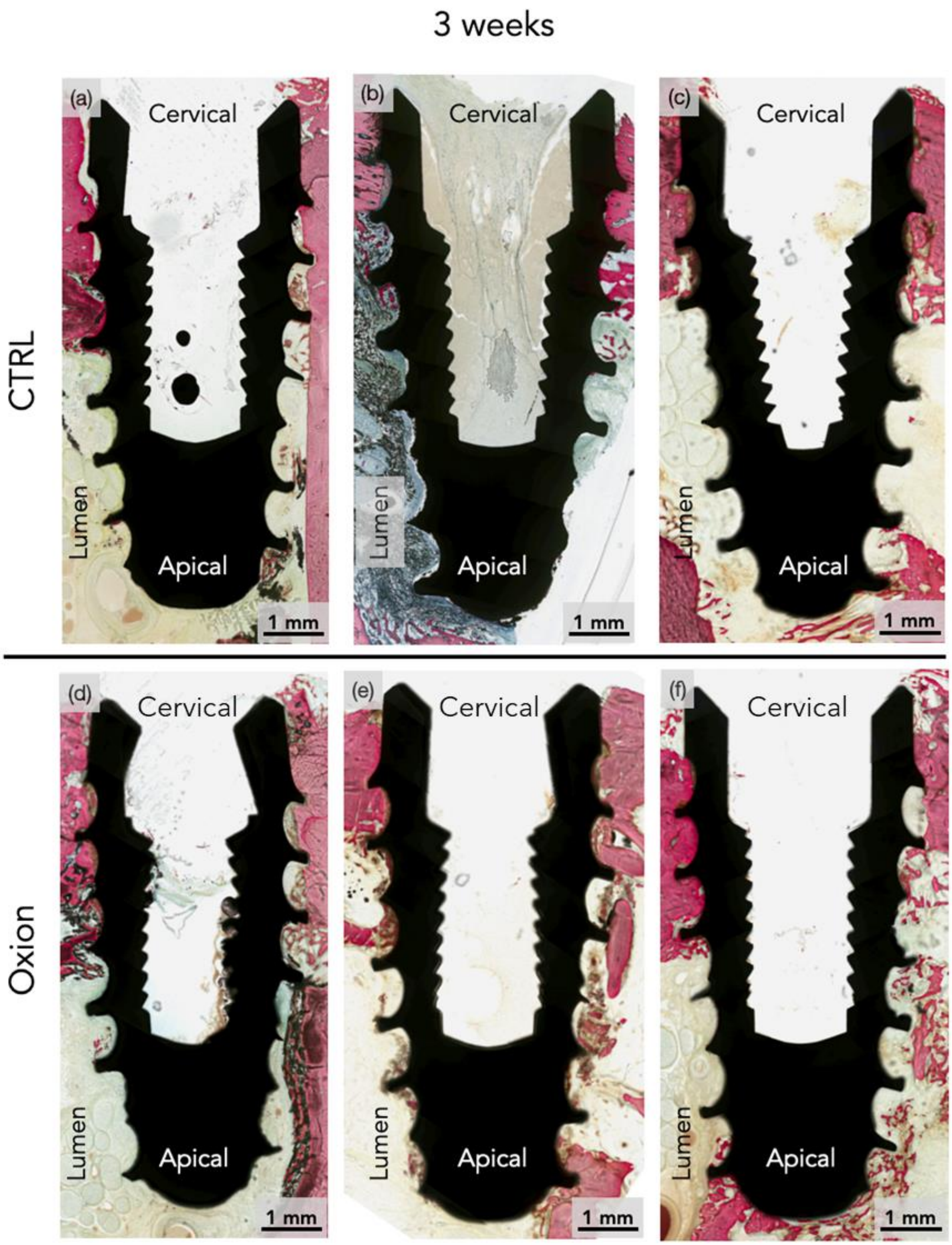

Supplementary Figure S1: Histological overviews of different samples from the (a-c) CTRL and (d-f) Oxion groups at 3 weeks showing calcified tissue in red and implant in black.

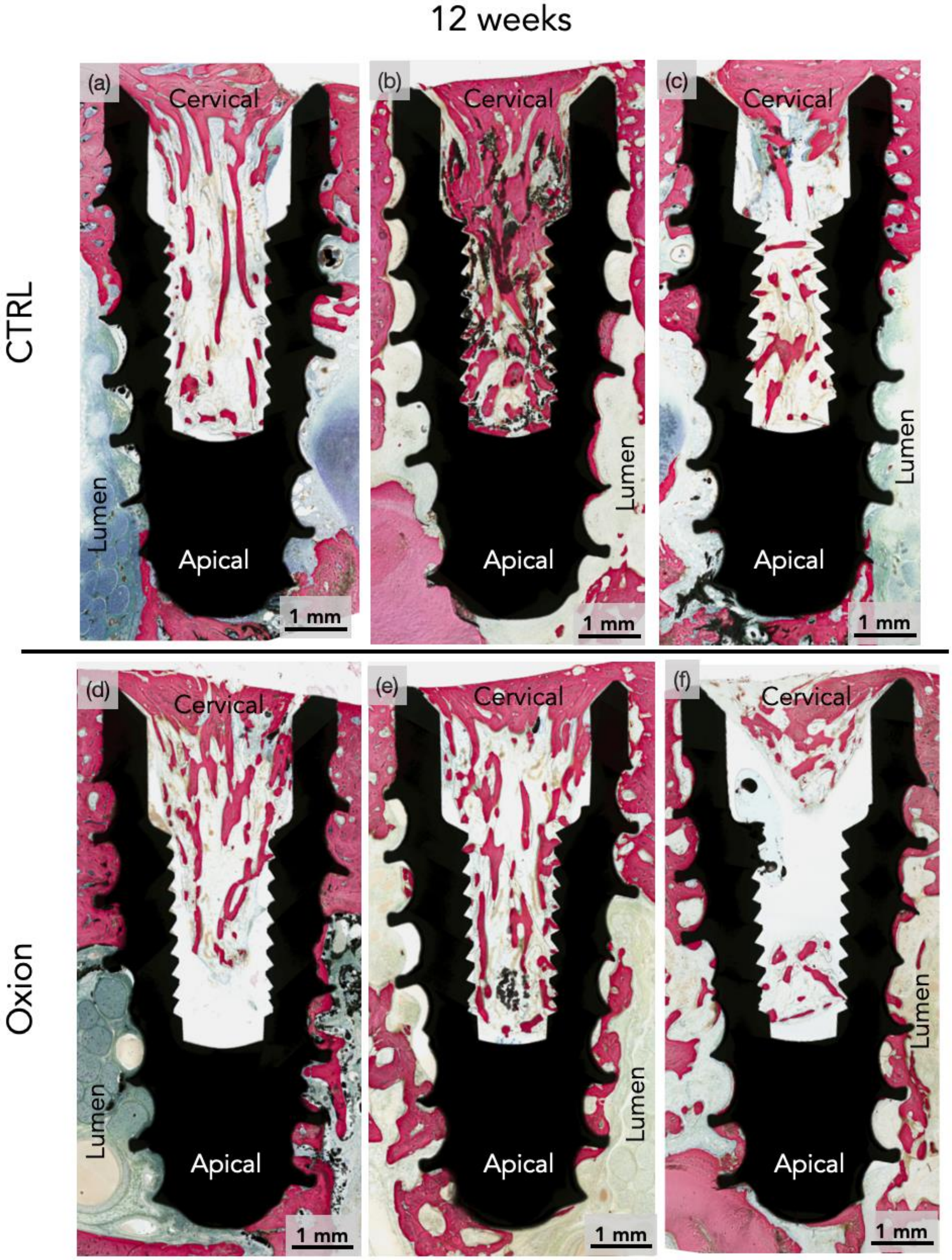

Supplementary Figure S2: Histological overviews of different samples from the (a-c) CTRL and (d-f) Oxion groups at 12 weeks showing calcified tissue in red and implant in black.
